# Case of Ascites, Treated by Introduction of Drainage Tube

**Published:** 1874-08

**Authors:** J. M. West

**Affiliations:** Springfield, Ill.


ARTICLE III.—*Case of Ascites, treated by the Introduction of a Drainage Tube.* By J. M. WEST, M.D., Springfield, Ill.

For the purpose of calling the attention of the profession to a somewhat novel method of treating a class of abdominal dropsies, the following case is reported:

A German farmer, aged sixty-six years, bachelor, residing some twenty miles from my office, called for advice and treatment. Has ascites of two years' standing; has had treatment, which was only palliative; could not detect either cardiac, renal or hepatic disease to be the cause of the dropsy, and hence concluded that there existed in this case a secretory action of the peritoneum above the capacity of the absorbents to remove. For the purpose of removing the accumulation now existing, tapped the patient on the 21st of April, 1873, removing three gallons of fluid. He was then put on the usual remedies for the purpose of preventing a reaccumulation, but without success, since on the 29th it became necessary to tap again. On the 10th of May was obliged to resort to the same process. Again on the 16th, in each case removing about three gallons of fluid.

It was observed, after being tapped, that for several days the patient's appetite was good; he slept well, and seemed to recuperate rapidly, until the distention appeared to interfere with the digestive and assimilative functions, then he rapidly lost ground again. A casual remark in the presence of the patient in reference to a drainage tube, set his wits to work, by which he came to the conclusion that if a tube was inserted in his abdomen for the escape of fluid, there would then remain nothing to prevent his speedy recovery. The danger of such practice was pointed out to him, but he did not seem to appreciate it; he seemed, as time went on, and other measures failed, only the more importunate to have it done. After consulting with Drs. Townsend and Phillips, of this city, and taking into account our belief that after a dropsy has existed for a time, the susceptibility of the peritoneum is very much lessened, it was determined to introduce the tube, which was done at last tapping. We believed that such a measure would relieve the embarrassed respiration and assist digestion, and a vigorous digestion and assimilation of plastic material would thereby enable him to surmount the difficulty.

I had made, a silver, spool-shaped tube for the occasion, corking it, and allowing the patient to remove the cork at will. The tube was allowed to remain ten days. At the expiration of that time, there being no further indication of fluid, and patient experiencing considerable pain, it was deemed best to remove it. The patient's abdomen feeling tense and lobulated, as well as tender to the touch, a few doses of opium and quietude set matters aright with an entire cure of our case. Up to this date, now for more than one year, the man has been entirely free from his dropsy. No doubt there was a sufficient amount of inflammatory action set up to change the relative balance between the secretive and absorptive action of the peritoneum, and probably to cause some adhesions between their opposing surfaces.

